# (4-Methyl­phen­yl)[2-(thio­phen-2-ylcarbon­yl)phen­yl]methanone

**DOI:** 10.1107/S1600536811032478

**Published:** 2011-08-17

**Authors:** V. Silambarasan, S. Sundaramoorthy, R. Sivasakthikumaran, A. K. MohanaKrishnan, D. Velmurugan

**Affiliations:** a CAS in Crystallography and Biophysics, University of Madras, Guindy Campus, Chennai 25, India; b Department of Organic Chemistry, University of Madras, Guindy Campus, Chennai 25, India

## Abstract

The crystal studied of the title compound, C_19_H_14_O_2_S, was an inversion twin with a 0.7 (1):0.3 (1) domain ratio. The central benzene ring makes dihedral angles of 63.31 (9) and 60.86 (9)°, respectively, with the 4-methyl­phenyl and thio­phene rings. In the crystal, mol­ecules are linked by weak inter­molecular C—H⋯O hydrogen bonds and S⋯π [3.609 (3) Å] inter­actions.

## Related literature

For the biological activity of thiophene derivatives, see: Bonini *et al.* (2005 ▶); Brault *et al.* (2005 ▶); Isloora *et al.* (2010 ▶); Xia *et al.* (2010 ▶). For related structures, see: Ranjith *et al.* (2011 ▶); Dufresne & Skene (2010 ▶).
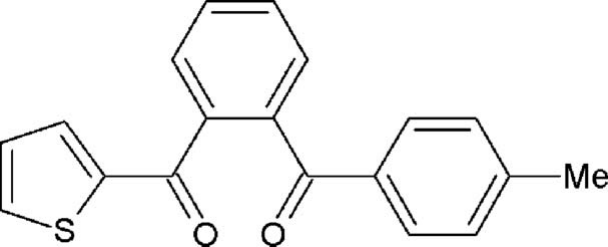


## Experimental

### 

#### Crystal data

C_19_H_14_O_2_S
*M* *_r_* = 306.36
Orthorhombic, 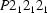

*a* = 6.8748 (2) Å
*b* = 13.3291 (4) Å
*c* = 16.8667 (5) Å
*V* = 1545.58 (8) Å^3^
*Z* = 4
Mo *K*α radiation
μ = 0.21 mm^−1^
*T* = 293 K
0.25 × 0.22 × 0.19 mm

#### Data collection

Bruker APEXII CCD area-detector diffractometer
Absorption correction: multi-scan (*SADABS*; Sheldrick, 1996 ▶) *T*_min_ = 0.948, *T*_max_ = 0.960
14787 measured reflections
3757 independent reflections
3073 reflections with *I* > 2σ(*I*)
*R*_int_ = 0.027

#### Refinement

*R*[*F* ^2^ > 2σ(*F* ^2^)] = 0.052
*wR*(*F* ^2^) = 0.154
*S* = 1.04
3757 reflections
195 parameters
H-atom parameters constrained
Δρ_max_ = 0.84 e Å^−3^
Δρ_min_ = −0.38 e Å^−3^
Absolute structure: Flack (1983 ▶), 1564 Friedel pairs
Flack parameter: 0.31 (11)

### 

Data collection: *APEX2* (Bruker, 2004 ▶); cell refinement: *SAINT* (Bruker, 2004 ▶); data reduction: *SAINT*; program(s) used to solve structure: *SHELXS97* (Sheldrick, 2008 ▶); program(s) used to refine structure: *SHELXL97* (Sheldrick, 2008 ▶); molecular graphics: *ORTEP-3* (Farrugia, 1997 ▶); software used to prepare material for publication: *SHELXL97* and *PLATON* (Spek, 2009 ▶).

## Supplementary Material

Crystal structure: contains datablock(s) global, I. DOI: 10.1107/S1600536811032478/lx2197sup1.cif

Structure factors: contains datablock(s) I. DOI: 10.1107/S1600536811032478/lx2197Isup2.hkl

Supplementary material file. DOI: 10.1107/S1600536811032478/lx2197Isup3.cml

Additional supplementary materials:  crystallographic information; 3D view; checkCIF report

## Figures and Tables

**Table 1:** Hydrogen-bond geometry (Å, °)

*D*—H⋯*A*	*D*—H	H⋯*A*	*D*⋯*A*	*D*—H⋯*A*
C9—H9⋯O2^i^	0.93	2.51	3.386 (3)	158

Symmetry code: (i) 
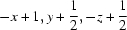
.

**Fig. 1.**
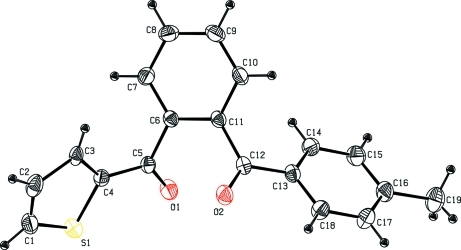
The structure of showing the atom–numbering scheme and intramolecular hydrogen bond. Displacement ellipsoids are drawn at the 30% probability level.

**Fig. 2.**
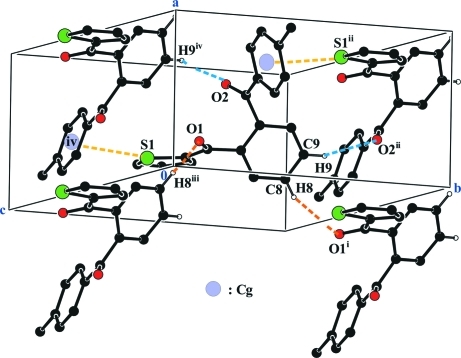
A view of the C—H···O and S···π interactions (dotted lines) in the crystal structure of the title compound. [Symmetry codes: (i) - x, y + 1/2 , - z + 1/2; (ii) - x + 1 , y + 1/2, - z + 1/2; (iii) - x , y -1/2, - z + 1/2; (iv) - x + 1, y - 1/2, - z + 1/2. ]

## supplementary crystallographic information

### Comment

Thiophene derivatives exhibit anti–HIVPR inhibitors (Bonini *et al.*, 2005) and anti–breast cancer(Brault *et al.*, 2005) activities. In addition, some of the benzo[*b*]thiophene derivatives show significant antimicrobial and anti–inflammatory activities (Isloora *et al.*, 2010). The thiophene derivates have been viewed as significant compounds for application in many fields (Xia *et al.*, 2010). Against this background, we report herein the crystal structure of the title compound.

The title compound crystallizes as the non–centrosymmetric space group *P*2_1_2_1_2_1_ in spite of having no asymmetric C atoms. The crystal studied was an inversion twin with a 0.7 (1):0.3 (1) domain ratio.

In the title compound (Fig. 1), The bond lengths and angles agree with those observed in other thiophene derivative (Ranjith *et al.*, 2011). The benzene ring makes the dihedral angles of 63.31 (9)° and 60.86 (9)°, respectively with the 4–methylphenyl and thiophene rings. The thiophene ring makes the dihedral angle of 75.04 (9)° with respect to 4–methylphenyl ring, it shows that both rings are almost perpendicular to each other.  The molecular packing (Fig. 2) is stabilized by weak intermolecular C—H···O hydrogen bonds; the first one between a benzene H atom and the O atom of the C═O unit (Table 1, C8—H8···O1^i^), and the second one between a benzene H atom and the O atom of the C═O unit (Table 1, C9—H9···O2^ii^). The molecular packing (Fig. 2) is further stabilized by a weak intermolecular S···π interaction the thiophene S atom and the 4–methylphenyl ring, with a S1···Cg^iv^ [3.609 (3) Å] (Cg is the centroid of the C13–C18 phenyl ring).

### Experimental

The furan 1 g was dissolved in THF. The weighted lead tetracetone (1.52 g, 1520 m mol) was added to the furan. Then it was refluxed at 343 K for 0.5 h. The reaction mixture was checked for TLC. Then the usual workup was done with brine solution and CHCl_3_ follwed by column chromatography (10% AcOEt/hexane) lead to the solution of the pure compound. Single crystals suitable for X–ray diffraction were obtained by slow evaporation of a solution of the title compound in ethyl acetate at room temperature.

### Refinement

All H atoms were fixed geometrically and allowed to ride on their parent C atoms, with C—H distances fixed in the range 0.93–0.97 Å with *U*_iso_(H) = 1.5*U*_eq_(C) for methyl H 1.2*U*_eq_(C) for other H atoms.

### Crystal data

**Table:**

C_19_H_14_O_2_S	*F*(000) = 640
*M**_r_* = 306.36	*D*_x_ = 1.317 Mg m^−^^3^
Orthorhombic, *P*2_1_2_1_2_1_	Mo *K*α radiation, λ = 0.71073 Å
Hall symbol: P 2ac 2ab	Cell parameters from 3758 reflections
*a* = 6.8748 (2) Å	θ = 2.0–28.2°
*b* = 13.3291 (4) Å	µ = 0.21 mm^−^^1^
*c* = 16.8667 (5) Å	*T* = 293 K
*V* = 1545.58 (8) Å^3^	Block, white crystalline
*Z* = 4	0.25 × 0.22 × 0.19 mm

### Data collection

**Table:**

Bruker APEXII CCD area-detector diffractometer	3757 independent reflections
Radiation source: fine-focus sealed tube	3073 reflections with *I* > 2σ(*I*)
graphite	*R*_int_ = 0.027
ω and φ scans	θ_max_ = 28.2°, θ_min_ = 2.0°
Absorption correction: multi-scan (*SADABS*; Sheldrick, 1996)	*h* = −9→8
*T*_min_ = 0.948, *T*_max_ = 0.960	*k* = −17→17
14787 measured reflections	*l* = −21→22

### Refinement

**Table:**

Refinement on *F*^2^	Secondary atom site location: difference Fourier map
Least-squares matrix: full	Hydrogen site location: difference Fourier map
*R*[*F*^2^ > 2σ(*F*^2^)] = 0.052	H-atom parameters constrained
*wR*(*F*^2^) = 0.154	*w* = 1/[σ^2^(*F*_o_^2^) + (0.0975*P*)^2^ + 0.2738*P*] where *P* = (*F*_o_^2^ + 2*F*_c_^2^)/3
*S* = 1.04	(Δ/σ)_max_ < 0.001
3757 reflections	Δρ_max_ = 0.84 e Å^−^^3^
195 parameters	Δρ_min_ = −0.38 e Å^−^^3^
0 restraints	Absolute structure: Flack (1983), 1564 Friedel pairs
Primary atom site location: structure-invariant direct methods	Flack parameter: 0.31 (11)

### Special details

**Table:**

Geometry. All esds (except the esd in the dihedral angle between two l.s. planes) are estimated using the full covariance matrix. The cell esds are taken into account individually in the estimation of esds in distances, angles and torsion angles; correlations between esds in cell parameters are only used when they are defined by crystal symmetry. An approximate (isotropic) treatment of cell esds is used for estimating esds involving l.s. planes.
Refinement. Refinement of F^2^ against ALL reflections. The weighted R-factor wR and goodness of fit S are based on F^2^, conventional R-factors R are based on F, with F set to zero for negative F^2^. The threshold expression of F^2^ > 2sigma(F^2^) is used only for calculating R-factors(gt) etc. and is not relevant to the choice of reflections for refinement. R-factors based on F^2^ are statistically about twice as large as those based on F, and R- factors based on ALL data will be even larger.

### Fractional atomic coordinates and isotropic or equivalent isotropic displacement parameters (Å^2^)

**Table:**

	*x*	*y*	*z*	*U*_iso_*/*U*_eq_	
C1	0.1784 (5)	0.1736 (3)	0.51343 (18)	0.0572 (8)	
H1	0.1693	0.1232	0.5514	0.069*	
C2	0.1844 (4)	0.2720 (2)	0.53175 (14)	0.0457 (4)	
H2	0.1790	0.2949	0.5838	0.055*	
C3	0.2000 (4)	0.3398 (2)	0.46487 (14)	0.0457 (4)	
H3	0.2065	0.4095	0.4665	0.055*	
C4	0.2035 (4)	0.27597 (17)	0.39395 (13)	0.0364 (5)	
C5	0.2388 (3)	0.30681 (16)	0.31244 (13)	0.0336 (5)	
C6	0.2404 (4)	0.41697 (16)	0.29365 (12)	0.0335 (5)	
C7	0.0825 (4)	0.4758 (2)	0.31315 (15)	0.0438 (6)	
H7	−0.0201	0.4487	0.3420	0.053*	
C8	0.0768 (5)	0.5760 (2)	0.28959 (17)	0.0536 (7)	
H8	−0.0296	0.6157	0.3027	0.064*	
C9	0.2277 (5)	0.61580 (19)	0.24717 (16)	0.0544 (7)	
H9	0.2235	0.6828	0.2318	0.065*	
C10	0.3856 (5)	0.55782 (18)	0.22697 (15)	0.0462 (6)	
H10	0.4872	0.5859	0.1981	0.055*	
C11	0.3943 (4)	0.45704 (16)	0.24953 (13)	0.0347 (5)	
C12	0.5750 (4)	0.39723 (16)	0.23542 (13)	0.0353 (5)	
C13	0.6576 (4)	0.39061 (17)	0.15393 (14)	0.0370 (5)	
C14	0.5507 (4)	0.41308 (19)	0.08690 (16)	0.0439 (6)	
H14	0.4223	0.4340	0.0920	0.053*	
C15	0.6323 (5)	0.4049 (2)	0.01220 (15)	0.0525 (7)	
H15	0.5573	0.4191	−0.0323	0.063*	
C16	0.8252 (5)	0.3755 (2)	0.00285 (15)	0.0519 (7)	
C17	0.9301 (5)	0.3518 (2)	0.06999 (17)	0.0543 (7)	
H17	1.0586	0.3311	0.0650	0.065*	
C18	0.8480 (4)	0.3583 (2)	0.14457 (16)	0.0463 (6)	
H18	0.9211	0.3409	0.1889	0.056*	
C19	0.9150 (7)	0.3703 (3)	−0.07938 (19)	0.0795 (11)	
H19A	0.8544	0.3174	−0.1091	0.119*	
H19B	1.0519	0.3572	−0.0748	0.119*	
H19C	0.8955	0.4331	−0.1062	0.119*	
O1	0.2661 (3)	0.24586 (12)	0.25971 (10)	0.0490 (5)	
O2	0.6549 (3)	0.35775 (15)	0.29111 (10)	0.0483 (5)	
S1	0.18865 (12)	0.15059 (5)	0.41576 (4)	0.0541 (2)	

### Atomic displacement parameters (Å^2^)

**Table:**

	*U*^11^	*U*^22^	*U*^33^	*U*^12^	*U*^13^	*U*^23^
C1	0.0465 (16)	0.0748 (19)	0.0504 (16)	0.0050 (15)	0.0071 (13)	0.0224 (14)
C2	0.0344 (9)	0.0752 (12)	0.0275 (7)	0.0050 (9)	0.0001 (7)	0.0048 (8)
C3	0.0344 (9)	0.0752 (12)	0.0275 (7)	0.0050 (9)	0.0001 (7)	0.0048 (8)
C4	0.0339 (12)	0.0403 (11)	0.0351 (11)	−0.0001 (10)	0.0018 (10)	0.0025 (9)
C5	0.0315 (12)	0.0366 (10)	0.0327 (11)	−0.0048 (9)	0.0016 (9)	0.0011 (8)
C6	0.0402 (13)	0.0333 (10)	0.0271 (10)	−0.0022 (9)	−0.0007 (9)	−0.0001 (8)
C7	0.0419 (15)	0.0500 (13)	0.0394 (13)	0.0057 (12)	0.0032 (11)	0.0017 (11)
C8	0.0650 (19)	0.0483 (14)	0.0476 (14)	0.0202 (14)	0.0050 (14)	−0.0002 (12)
C9	0.084 (2)	0.0355 (11)	0.0438 (14)	0.0101 (13)	0.0057 (15)	0.0066 (10)
C10	0.0640 (18)	0.0370 (11)	0.0378 (12)	−0.0016 (12)	0.0100 (12)	0.0066 (10)
C11	0.0431 (13)	0.0346 (10)	0.0265 (10)	−0.0024 (9)	−0.0003 (9)	0.0005 (8)
C12	0.0379 (12)	0.0375 (11)	0.0304 (11)	−0.0072 (9)	0.0002 (9)	0.0015 (9)
C13	0.0448 (14)	0.0351 (10)	0.0311 (11)	−0.0057 (10)	0.0025 (10)	−0.0011 (9)
C14	0.0475 (15)	0.0488 (13)	0.0352 (12)	0.0052 (11)	−0.0001 (11)	0.0020 (11)
C15	0.066 (2)	0.0595 (16)	0.0317 (13)	0.0077 (14)	−0.0008 (12)	0.0007 (11)
C16	0.073 (2)	0.0504 (14)	0.0327 (12)	0.0000 (15)	0.0097 (13)	−0.0047 (10)
C17	0.0479 (15)	0.0639 (17)	0.0512 (16)	0.0054 (14)	0.0120 (13)	−0.0039 (14)
C18	0.0435 (15)	0.0566 (14)	0.0386 (13)	0.0029 (13)	−0.0020 (11)	−0.0016 (11)
C19	0.107 (3)	0.091 (2)	0.0410 (16)	0.014 (2)	0.0258 (18)	−0.0050 (16)
O1	0.0730 (14)	0.0372 (8)	0.0369 (8)	−0.0093 (8)	0.0091 (9)	−0.0068 (7)
O2	0.0488 (11)	0.0629 (11)	0.0333 (9)	0.0044 (9)	0.0005 (8)	0.0104 (8)
S1	0.0591 (4)	0.0503 (4)	0.0529 (4)	0.0018 (3)	0.0079 (3)	0.0118 (3)

### Geometric parameters (Å, °)

**Table:**

C1—C2	1.348 (5)	C10—C11	1.397 (3)
C1—S1	1.677 (3)	C10—H10	0.9300
C1—H1	0.9300	C11—C12	1.495 (3)
C2—C3	1.449 (4)	C12—O2	1.209 (3)
C2—H2	0.9300	C12—C13	1.490 (3)
C3—C4	1.468 (3)	C13—C14	1.381 (4)
C3—H3	0.9300	C13—C18	1.387 (4)
C4—C5	1.455 (3)	C14—C15	1.383 (4)
C4—S1	1.714 (2)	C14—H14	0.9300
C5—O1	1.219 (3)	C15—C16	1.392 (5)
C5—C6	1.502 (3)	C15—H15	0.9300
C6—C7	1.379 (3)	C16—C17	1.379 (4)
C6—C11	1.399 (3)	C16—C19	1.520 (4)
C7—C8	1.393 (4)	C17—C18	1.381 (4)
C7—H7	0.9300	C17—H17	0.9300
C8—C9	1.367 (4)	C18—H18	0.9300
C8—H8	0.9300	C19—H19A	0.9600
C9—C10	1.375 (4)	C19—H19B	0.9600
C9—H9	0.9300	C19—H19C	0.9600
			
C2—C1—S1	113.7 (2)	C10—C11—C12	120.3 (2)
C2—C1—H1	123.2	C6—C11—C12	120.61 (19)
S1—C1—H1	123.2	O2—C12—C13	121.2 (2)
C1—C2—C3	115.5 (3)	O2—C12—C11	119.1 (2)
C1—C2—H2	122.2	C13—C12—C11	119.7 (2)
C3—C2—H2	122.2	C14—C13—C18	118.4 (2)
C2—C3—C4	105.9 (2)	C14—C13—C12	122.7 (2)
C2—C3—H3	127.0	C18—C13—C12	118.9 (2)
C4—C3—H3	127.0	C13—C14—C15	120.9 (3)
C5—C4—C3	127.5 (2)	C13—C14—H14	119.6
C5—C4—S1	119.21 (17)	C15—C14—H14	119.6
C3—C4—S1	112.90 (18)	C14—C15—C16	120.8 (3)
O1—C5—C4	121.8 (2)	C14—C15—H15	119.6
O1—C5—C6	119.7 (2)	C16—C15—H15	119.6
C4—C5—C6	118.5 (2)	C17—C16—C15	118.0 (3)
C7—C6—C11	120.3 (2)	C17—C16—C19	121.8 (3)
C7—C6—C5	120.0 (2)	C15—C16—C19	120.2 (3)
C11—C6—C5	119.4 (2)	C16—C17—C18	121.3 (3)
C6—C7—C8	119.9 (3)	C16—C17—H17	119.3
C6—C7—H7	120.0	C18—C17—H17	119.3
C8—C7—H7	120.0	C17—C18—C13	120.6 (3)
C9—C8—C7	120.0 (3)	C17—C18—H18	119.7
C9—C8—H8	120.0	C13—C18—H18	119.7
C7—C8—H8	120.0	C16—C19—H19A	109.5
C8—C9—C10	120.7 (2)	C16—C19—H19B	109.5
C8—C9—H9	119.7	H19A—C19—H19B	109.5
C10—C9—H9	119.7	C16—C19—H19C	109.5
C9—C10—C11	120.4 (3)	H19A—C19—H19C	109.5
C9—C10—H10	119.8	H19B—C19—H19C	109.5
C11—C10—H10	119.8	C1—S1—C4	92.00 (14)
C10—C11—C6	118.6 (2)		
			
S1—C1—C2—C3	−0.4 (4)	C5—C6—C11—C12	−12.7 (3)
C1—C2—C3—C4	0.3 (3)	C10—C11—C12—O2	122.4 (3)
C2—C3—C4—C5	−172.8 (2)	C6—C11—C12—O2	−50.0 (3)
C2—C3—C4—S1	0.0 (3)	C10—C11—C12—C13	−55.2 (3)
C3—C4—C5—O1	169.2 (2)	C6—C11—C12—C13	132.4 (2)
S1—C4—C5—O1	−3.1 (3)	O2—C12—C13—C14	164.1 (2)
C3—C4—C5—C6	−11.0 (4)	C11—C12—C13—C14	−18.3 (3)
S1—C4—C5—C6	176.69 (18)	O2—C12—C13—C18	−14.3 (3)
O1—C5—C6—C7	124.5 (3)	C11—C12—C13—C18	163.3 (2)
C4—C5—C6—C7	−55.3 (3)	C18—C13—C14—C15	−0.8 (4)
O1—C5—C6—C11	−49.3 (3)	C12—C13—C14—C15	−179.3 (2)
C4—C5—C6—C11	131.0 (2)	C13—C14—C15—C16	−1.2 (4)
C11—C6—C7—C8	−0.7 (4)	C14—C15—C16—C17	2.0 (4)
C5—C6—C7—C8	−174.4 (2)	C14—C15—C16—C19	−177.8 (3)
C6—C7—C8—C9	0.0 (4)	C15—C16—C17—C18	−0.9 (5)
C7—C8—C9—C10	0.3 (5)	C19—C16—C17—C18	178.9 (3)
C8—C9—C10—C11	0.0 (4)	C16—C17—C18—C13	−1.1 (5)
C9—C10—C11—C6	−0.7 (4)	C14—C13—C18—C17	1.9 (4)
C9—C10—C11—C12	−173.2 (3)	C12—C13—C18—C17	−179.6 (3)
C7—C6—C11—C10	1.0 (3)	C2—C1—S1—C4	0.3 (3)
C5—C6—C11—C10	174.8 (2)	C5—C4—S1—C1	173.2 (2)
C7—C6—C11—C12	173.5 (2)	C3—C4—S1—C1	−0.2 (2)

### Hydrogen-bond geometry (Å, °)

**Table:**

*D*—H···*A*	*D*—H	H···*A*	*D*···*A*	*D*—H···*A*
C9—H9···O2^i^	0.93	2.51	3.386 (3)	158.

Symmetry codes: (i) −*x*+1, *y*+1/2, −*z*+1/2.
